# Opportunities of Reducing the Energy Consumption of Seawater Reverse Osmosis Desalination by Exploiting Salinity Gradients

**DOI:** 10.3390/membranes12111045

**Published:** 2022-10-26

**Authors:** Miguel-Ángel Aumesquet-Carreto, Bartolomé Ortega-Delgado, Lourdes García-Rodríguez

**Affiliations:** 1 Department of Energy Engineering, University of Seville, Escuela Técnica Superior de Ingeniería, Camino de los Descubrimientos s/n, 41092 Sevilla, Spain; 2 Plataforma Solar de Almería, CIEMAT, Ctra. de Senés s/n, 04200 Almería, Spain

**Keywords:** seawater desalination, pressure-retarded osmosis, salinity gradients, reverse osmosis, energy efficiency, industrial water reuse

## Abstract

This work presents a performance assessment of three seawater reverse osmosis—pressure-retarded osmosis (SWRO-PRO) hybrid schemes for energy consumption reduction in seawater desalination applications by using an external low salinity water source. For comparison purposes, another arrangement based on the conventional SWRO process combined with brackish water RO (BWRO) and desalination was analyzed. Reverse osmosis system analysis software environments were used to select the best SWRO configuration and operating conditions. A purposely developed model was used to evaluate the PRO system. Two different cases were assessed depending on the origin of the external low-salinity resource for the PRO process: industrial wastewater and urban treated wastewater. In the case of the industrial wastewater, due to regulations on wastewater reclamation, the best arrangement would be the first SWRO-PRO scheme which was analyzed with a specific energy consumption of 1.54 kWh/m^3^. If urban treated wastewater is available as an external resource, the results obtained show that this scheme, leading to the minimum specific energy consumption of 1.46 kWh/m^3^, is the conventional SWRO combined with BWRO. Therefore, hybrid SWRO-PRO systems are recommended to reduce the specific energy consumption of seawater desalination if an industrial wastewater source with low osmotic pressure is available.

## 1. Introduction

Water scarcity represents an increasing concern in many countries worldwide and is one of the major threats that could face the world population in the next decades. According to the United Nations [1], up to 26% of the world’s population lacked secure drinking water in 2020, while 2.3 billion people resided in countries suffering from water stress. On the other hand, approximately 96.5% of the total water on Earth is saline water from the oceans [2]; hence, seawater desalination technology seems a suitable option to mitigate drinking water scarcity. However, seawater desalination is still an energy-intensive process compared to other solutions: while the conventional treatment of surface and groundwater requires about 0.37 and 0.48 kWh/m^3^, respectively, in the case of seawater, it is 2.5 kWh/m^3^ or even more [3]. Nevertheless, seawater desalination is still one of the best options to satisfy the global freshwater demand due to the fact that surface water resources are scarce.

Reverse osmosis (RO) is the dominant technology in the seawater desalination market worldwide due to its lower energy consumption compared with thermal processes (such as, multi-effect distillation and multistage flash distillation) [4]. The practical thermodynamic limit of seawater reverse osmosis (SWRO), in terms of specific energy consumption (SEC), is about 1.6 kWh/m^3^ for desalting saltwater of 35 g/L with a 50% recovery [5]. Even though the real SEC of the single-stage SWRO process is near this thermodynamic limit (<3 kWh/m^3^), the total energy consumption of SWRO plants is higher than 3 kWh/m^3^ [6] due to seawater pre-treatment and post-treatment, system inefficiencies, etc. In this context, the hybridization of SWRO with technologies powered by renewable energy can help to further decrease the SEC of the conventional SWRO process.

Salinity gradient-harnessing has become attractive in recent years mainly due to the so far unexploited high potential of this source of energy, which is estimated to be 2000 TWh/year [7]. There are two main technologies able to extract this kind of energy: pressure retarded osmosis (PRO) and reverse electrodialysis (RED). Both processes extract energy from the controlled mixing of two solutions with different salt concentrations. Nevertheless, in general, the PRO process provides a higher efficiency and power density than RED [8]. In the PRO process, two salt solutions, a high-concentrated solution (draw solution, DS) and a low-concentrated solution (feed solution, FS), are separated by a semipermeable membrane. In these conditions, a water flux passes spontaneously from the diluted solution to the concentrated solution through the membrane. If an external pressure (lower than the osmotic pressure) is applied to the DS, energy can be recovered from the pressurized water volume and transferred.

The potential of the PRO process as a sustainable and emission-free energy source for power production or as an energy recovery system has been highlighted by several authors from the middle of the twentieth century [9,10,11,12,13,14,15,16,17,18]. Gonzales et al. [19] and Jiao et al. [20] assessed the prospects of PRO for electricity generation. The latter pointed out that the exploitation of seawater and river water mixing at estuaries proved to be infeasible; however, hypersaline sources such as the Red Sea should be studied. On the contrary, Aseffa et al. [21] recommended an optimized PRO system that was able to produce 0.07 kWh/m^3^ of net power from the mixing of river water and seawater, thus resulting in 12.34 TWh/year from the major rivers of India.

Regarding PRO membranes, the main bottlenecks rely on biofouling and limited power density. The biofouling tendency can be improved by controlling the reverse solute diffusion from the draw solution by using a suitable selection of solutes and the concentration of the draw solution [22]. In addition, outstanding experimental results concerning integrally-skinned asymmetric membranes have achieved between 3.6 and 10 times better power density than commercial PRO modules, as a key aspect for the further development of PRO systems [23].

Hybrid schemes between SWRO and PRO can help to minimize the specific energy consumption of the seawater desalination process. The concentrated brine rejected from the RO process can be used as the draw solution for the PRO, and the power produced by the PRO unit could reduce the pumping requirements of the SWRO desalination. The hybridization of SWRO processes with PRO to decrease specific energy consumption has been widely reported in the recent literature. A significant number of papers have been published on this topic in recent years. A brief resume is detailed in Table 1, focusing on the design configurations to integrate SWRO-PRO processes.

To sum up, the literature survey points out three different opportunities for coupling the SWRO desalination to the PRO process depending on the role of the PRO system, namely, supplying an electricity demand, providing the pretreatment of seawater feed to the SWRO desalination and obtaining additional energy recovery from the brine flow. Within this context, this paper focuses on the latter option.

This paper investigates the reduction of specific energy consumptions in the SWRO desalination process by integrating pressure-retarded osmosis, providing that a low osmotic pressure flow is available. With this objective, Sarp et al. [37] proposed relevant SWRO-PRO configurations in patent no. US 9,428,406 B2. However, no data on specific energy consumptions were provided. This work has been selected, among the revised literature, to analyze the performance improvement of the SWRO-PRO hybrid scheme and the energy consumption decrease by the recovering of osmotic energy from the rejected brine of the SWRO process. Specifically, the SEC of three different SWRO-PRO configurations proposed in [37] have been assessed. Unlike the methodology adopted in the literature in order to assess the improvements of SWRO-PRO configurations, a fourth configuration, based on the conventional scheme SWRO combined with BWRO, has also been evaluated. Thus, not only the treatment of the seawater feed but also the treatment of the PRO feed (a low concentration flow) has been considered in order to make a fair comparison between the different arrangements. Two of the layouts assessed in [38] are quite similar to those analyzed in reference [32] and proposed in [35].

A reverse osmosis system design software [38] was used to select the SWRO membrane elements and configurations (the number of pressure vessels and elements) which best fit the conditions provided for in the patent. The values of the main operating variables (a mass flow rate, pressure, and concentration) for each stream of the patent were determined using the SWRO membrane configuration obtained in the design software and the data provided in the aforementioned reference. Finally, two different cases were evaluated depending on the origin of the external resource and the possibilities of water reclamation: the industrial wastewater or treated urban wastewater.

## 2. Materials and Methods

### 2.1. Description of the System

Three different arrangements for the hybridization of the SWRO desalination and PRO processes have been selected from the existing literature [37]. They are referred to as configurations A, B, and C. These three integrating schemes are detailed in the following subsections. For all the arrangements, according to the mentioned reference, a feed seawater flow rate of 100 m^3^/h is assumed with a concentration of 40 g/L at 28 °C and a 50% recovery rate for the RO unit.

#### 2.1.1. Configuration A

**Figure 1:**
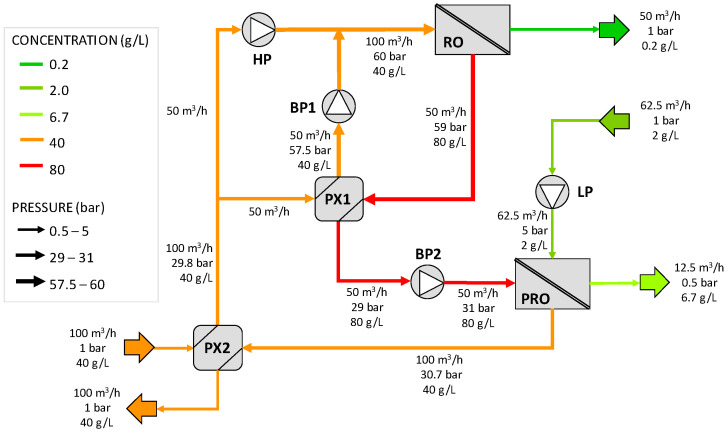
Schematic diagram of the first SWRO-PRO arrangement (configuration A) proposed in the analyzed patent. HP = high-pressure pump, LP = low-pressure pump, BP = booster pump, PX = pressure exchanger. Redrawn from [37].

The first configuration, depicted in Figure 1, is composed of an RO unit, a PRO system, two pressure exchangers (PX1 and PX2), a high-pressure pump (HP), a low-pressure pump (LP), and two booster pumps (BP1 and BP2). Note that the second pressure exchanger, PX2, is associated with the PRO process and is used to recover the pressure energy from the volume of water transferred within the PRO system. The inlet seawater, at ambient pressures, enters the pressure exchanger PX2, where it is pressurized up to 29.8 bar by the diluted brine (draw solution) from the PRO process. The pressurized inlet seawater is then divided into two equal streams: the first enters the pressure exchanger PX1, where it gains pressure (up to 57.5 bar) due to the energy partially transferred by the RO brine outlet. The second stream is directed to the high-pressure pumps (HP), where it is pressurized up to the operating pressure of the desalination process (60 bar). This stream is mixed with the feed outlet of the PX1 and, after evening, its pressures through the booster pump BP1. This mixed stream (RO feed) enters the core of the RO unit, obtaining the permeate (product) and the rejected brine. This RO brine constitutes the draw solution of the PRO process. As this stream contains high-pressure energy (59 bar), it is used to pressurize the RO feedwater in the PX1. Before entering the PRO unit, it is slightly pressurized by the booster pump BP2 from 29 to 31 bar. On the other side, the dilute solution for the PRO unit, which is assumed to be treated wastewater, is pressurized from the atmospheric pressure to 5 bar in the LP. In this configuration, an increase in the volumetric flow rate of the draw solution of 100% is assumed for the PRO process. Part of the inlet dilute solution permeates the pressurized draw solution, and the diluted draw solution at 30.7 bar enters the PX2, releasing its pressure energy to the feedwater. In this way, the PRO unit is used for reducing the pumping requirements of the RO desalination process and the concentration of the brine disposal.

#### 2.1.2. Configuration B

**Figure 2:**
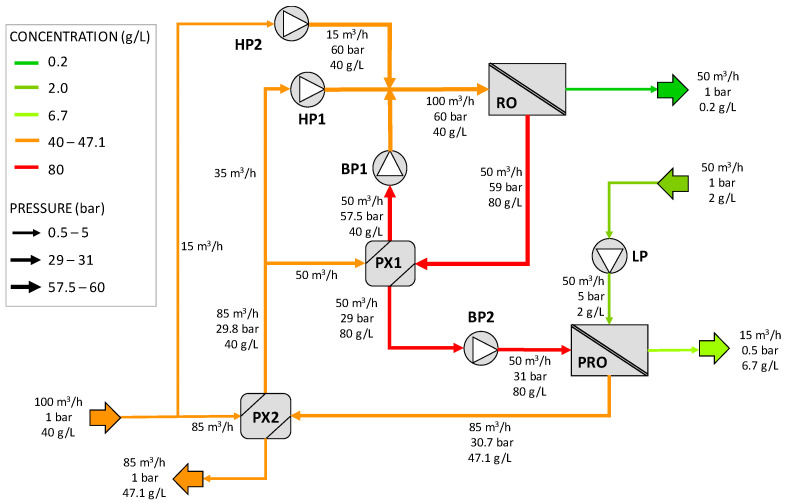
Schematic diagram of the second SWRO-PRO arrangement proposed in the patent (configuration B). HP = high-pressure pump, LP = low-pressure pump, BP = booster pump, PX = pressure exchanger. Redrawn from [37].

The second SWRO-PRO scheme proposed in the patent as presented in Figure 2. The feed seawater, at an ambient pressure, is divided into two streams: the first one (15 m^3^/h) is directed to the high-pressure RO pumps (HP2), where it is pressurized up to 60 bar. The second stream (85 m^3^/h) is driven to the pressure exchanger PX2, increasing its pressure to 29.8 bar by partially recovering pressure from the diluted draw solution exiting in the PRO unit. At the outlet, this stream is divided in two: part of it (35 m^3^/h) is directed to the high-pressure pumps (HP1) of the RO unit, and the rest (50 m^3^/h) to a pressure exchanger (PX1) used to recover the pressure energy from the brine produced in the SWRO process. In PX1, this stream increases its pressure up to 57.5 bar and, using a booster pump (BP1), evens its pressure to the operating pressure of the RO process (60 bar). The three streams at 60 bars are then mixed and driven to the RO unit, where the permeate is produced, and the concentrated brine is used to recover the pressure energy in PX1.

After passing through the PX1, the RO brine at 29 bar increases its pressure in the low booster pump (BP2) up to 31 bar and is used as a draw solution at the PRO unit. An increase in the volumetric flow rate of the draw solution of 70% for the PRO process is assumed. On the other side, the dilute feed solution of the PRO unit increases its pressure in the LP pump by up to 5 bar. Part of it permeates through the osmotic membranes and dilutes the draw solution, gaining pressure up by to 30.7 bar. This stream is used in PX2 to recover the pressure energy and increase the pressure of the feed stream. Finally, the diluted RO brine is rejected at an ambient pressure. Note that this configuration requires HP2 in addition to HP1, since the pressure exchanger operation requires similar flow rates for both streams. This is the reason for splitting the feedwater flow rate into 85 and 15 m^3^/h.

#### 2.1.3. Configuration C

**Figure 3:**
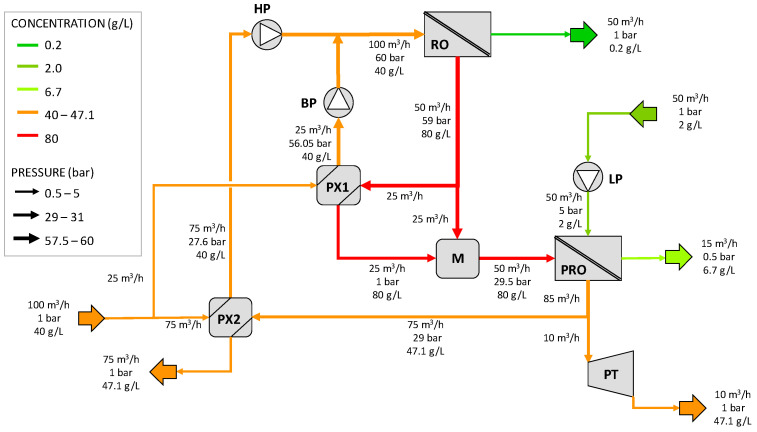
Schematic diagram of the third SWRO-PRO arrangement proposed in the patent (configuration C). HP = high-pressure pump, LP = low-pressure pump, BP = booster pump, PX = pressure exchanger, M = Mixer, PT = Pelton turbine. Redrawn from [37].

The third configuration proposed in [37] is depicted in Figure 3. This scheme is similar to the previous ones, although some differences can be observed. The feed seawater is split into two streams: a small part of this stream (25 m^3^/h) goes to the pressure exchanger of the RO unit, PX1, where it is pressurized up to 56.05 bar, and using the booster pump (BP), it evens the operating pressure of RO (60 bar). The other stream (75 m^3^/h) is pressurized in PX2 up to 27.6 bar before entering the HP pumps, where it is further pressurized to 60 bars. Both streams are then mixed at the inlet of the RO unit. At the outlet of the RO system, two streams are produced: the permeate flow and the rejected brine, part of which (25 m^3^/h) is used to recover pressure energy, and the other part (25 m^3^/h) of which is mixed in the mixer (M) with the diluted brine at the outlet of the PX1. Note that with respect to configuration B, the booster pump associated with this stream is eliminated. The resulting stream, at 29.5 bar, is the draw solution into the PRO unit. On the other side, the dilute feed solution of the PRO is pressurized in the LP pump by up to 5 bar before entering the PRO unit, and part of it permeates to the concentrated side, hence gaining pressure. The diluted draw solution at the outlet of the PRO unit at 29 bar is split into two streams: 75 m^3^/h is used in the PX2 to increase the pressure of the feed seawater, while the rest (10 m^3^/h) is driven to a Pelton turbine to obtain mechanical energy. This configuration is the only one that has a hydraulic turbine, and therefore, the power produced can be used for pumping, thereby reducing the SEC.

### 2.2. Methods

The starting point for the analysis consists of the selection of three configurations proposed in the literature to improve the energy performance of the SWRO desalination process, as described in Section 2.1. As a case base for allowing the assessment of the actual improvement, a conventional configuration of the SWRO desalination was considered and specifically designed to treat the same input flows, which are the same in all configurations:

- Low salinity stream: 62.5 m^3^/h; 1 bar; 2 g/L.
- High salinity stream: 100 m^3^/h; 1 bar; 40 g/L.

**Figure 4:**
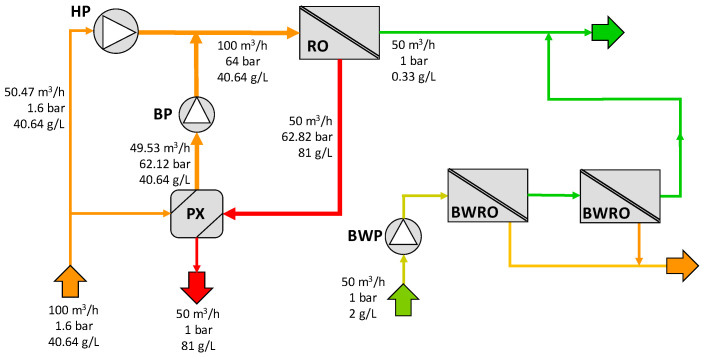
Base case configuration of the SWRO desalination process with BWRO (configuration D). HP = high-pressure pump, BWP = brackish water pump, PX = pressure exchanger.

Therefore, a seawater RO scheme with the same inputs as those described in the patent was defined (configuration D) to perform a fair performance comparison of the three SWRO-PRO configurations alongside the single SWRO desalination process. To make this standalone SWRO process similar to the other three configurations, an external brackish water stream was added, with a volumetric flow rate of 50 m^3^/h and a concentration of 2 g/L, in correspondence with the feedwater stream of the PRO process. This brackish water was desalinated with a two-pass brackish water reverse osmosis (BWRO) system (see Figure 4), which was modelled by using Q+ software [39].

Concerning the membrane rack in the SWRO desalination system, an RO design software environment [38] was used to perform the best selection for the design and operating parameters. The next section describes the analysis that was conducted on each component. In addition, concerning the PRO units, operating parameters reported by Sarp et al. [37] were assumed to be the initial hypothesis regardless of the design features of the system.

#### 2.2.1. Selection of the SWRO Design

The selection of the SWRO membrane elements was made with an RO design software environment [38]. The first step consisted of selecting the composition of the feed seawater. Mediterranean seawater with a salinity of 40.6 g/L and a temperature of 28 °C [40] was considered. The chemical composition of the seawater is presented in Table 2. SW30XLE-440i RO membranes from DOW FILMTEC^TM^, one-pass, and one-stage configuration were considered. Several configurations were tested to reach the desired specifications (50 m^3^/h permeate flow, 50% recovery), varying the pressure vessel (PV) number and the number of membrane elements within a PV, resulting in the best configuration of 13 PV with 7 elements each, in terms of the minimum specific energy consumption. The isentropic and mechanical efficiencies of the pumps were selected as 85% and 95%, respectively, a flow factor (a factor considering the fouling of the membranes, aging, pressure, and operation time) of 0.85, and in the case of the third configuration, a total efficiency of the Pelton turbine of 85%.

The results of the simulation carried out with the values above-mentioned, i.e., 13 PV and 7 elements each, are depicted in Table 3, following the results presented by Sarp et al. [37]. However, slight differences can be observed, including the feed flow rate pressure of 64 bar instead of 60 bar.

#### 2.2.2. PRO Model

**Figure 5:**
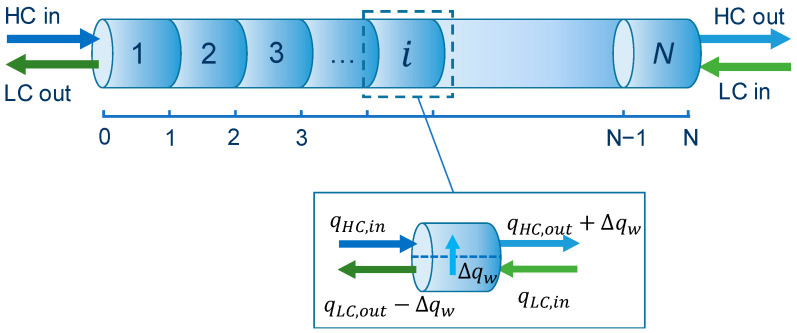
Scheme of the membrane and discretization. HC = High concentrated stream, LC = Low concentrated stream.

A simplified mathematical model has been developed for the PRO subsystem to validate the data provided in [37]. The model, implemented in the Microsoft Excel software environment, divides the membrane into *N* control volumes at steady-state conditions in which a given value of water flow is transferred through the corresponding membrane area (see Figure 5).

Water and salt mass balance equations are applied in each control volume, for the high-concentrated (HC) and the low-concentrated (LC) flows,

(1) $$q_{HC,i+1}=q_{HC,i}+\Delta q_{w}$$

(2) $$q_{LC,i}=q_{LC,i+1}-\Delta q_{w}$$

(3) $$q_{HC,i+1}\cdot S_{HC,i+1}=q_{HC,i}\cdot S_{HC,i}$$

(4) $$q_{LC,i}\cdot S_{LC,i}=q_{LC,i+1}\cdot S_{LC,i+1}$$

where $q_{HC}$ (kg/s) is the mass flow rate of the HC solution, $\Delta q_{w}$ (kg/s) is the mass flow rate of water passing through the membrane, $q_{LC}$ (kg/s) is the mass flow rate of the LC solution, and $S_{HC}$, $S_{LC}$ (kg/kg) are the salinity of the HC and LC solutions, respectively. Subscripts i and i+1 stand for the inlet and outlet of each control volume, respectively.

Note that an equal amount of water flow $\Delta q_{w}$ passing through each membrane element is assumed. The model neglects the detrimental effects of internal/external concentration polarizations and reverse salt flux. The thermophysical properties of seawater and feedwater are determined using the equations provided by Sharqawy et al. [41]. The driving force DF of the process is expressed by:

(5) $$DF=\Delta \Pi -\Delta p=\left(\Pi_{\mathrm{HC}}-\Pi_{LC}\right)-\left(p_{HC}-p_{LC}\right)$$

where ΔΠ (bar) is the osmotic pressure difference and Δp (bar) is the hydraulic pressure difference. The osmotic pressure Π (Pa) has been determined by Equation (6):

(6) $$\Pi =\phi \cdot \rho_{w}^{*}\cdot R\cdot T\cdot b_{s\pm }=2\cdot \phi \cdot \rho_{w}^{*}\cdot R\cdot T\cdot b_{s}$$

where ϕ (-) is the osmotic coefficient, $\rho_{w}^{*}$ (kg/m^3^) is the water density, R (J/(mol·K)) is the universal gas constant, T (K) is the temperature, and $b_{s}$ (mol/kg) is the molality of the solute.

For inputs, the model uses the flow rates, temperature, pressure, and salinity of the inlet streams (of HC and LC solutions) and the stream pressures at the outlet. For convenience, the volumetric flow rate of water transferred in the PRO has been determined to match the required amount needed in the PX.

**Figure 6:**
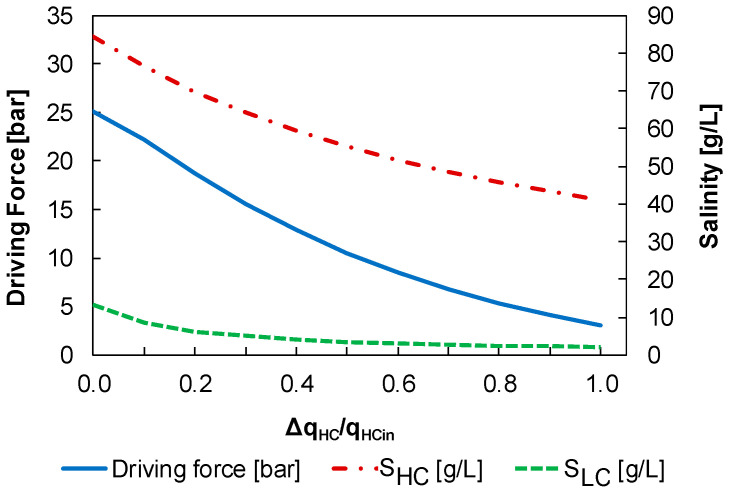
Driving force and salinity profiles as a function of the HC flow rate increase in configuration A, where $\Delta q_{HC}/q_{HC,in}$ means the ratio of the mass flow rate of water transferred Δ($q_{w}$) to the HC mass flow rate ($q_{HC,in}$).

Using this model, the driving force and salinity profiles of the HC and LC flows are determined along the membrane (see Figure 6). Note how the osmotic pressure difference between the HC and LC solutions decreases with the increase in the water flow from the LC solution to the HC solution.

A comparison between the values reported by the patent in the PRO process and the ones obtained by the model for each configuration A, B, and C are presented in Table 4, Table 5 and Table 6, respectively. The cells in grey indicate the input value for the model. As can be seen, the values obtained by the model are quite similar to those of the patent.

#### 2.2.3. Selection of the BWRO Design in the Base Case

The configuration corresponding to the base case includes a BWRO unit. The BWRO systems that were considered have two membrane passes, and therefore, the permeate crosses two membranes with a pore size of RO. Actual regulations on the reuse (or reclamation) of wastewater require two passes through a membrane type of forwarding osmosis, nanofiltration, pressure-retarded osmosis, or reverse osmosis. Therefore, this system would fulfill this requirement, and it would be suitable for desalting the feed stream for the PRO process of the patent in the case of treated wastewater. Specific energy consumption of 0.5 kWh/m^3^ for the BWRO process was obtained by using Q+ design software [39] with the following design and operating parameters:

- Brackish water: temperature, 28 °C; pH, 7.0; salinity, 2 g/L; individual components and corresponding mass fraction from reference [40].
- Design parameters: feed water, 50 m^3^/h, total recovery rate, 81%; pass 1 with 2 stages; pass 2 with 1 stage; pump isentropic efficiency, 85%, pump mechanical efficiency, 95%; energy recovery device, turbocharger with 90% efficiency.

Thus obtaining:

- Permeate: 40.5 m^3^/h.
- Specific energy consumption: 0.5 kWh/m^3^.

#### 2.2.4. Isobaric Chambers (Pressure Exchangers, PX)

There are two pressure exchangers in all three arrangements of the patent that were analyzed, with different inlet and outlet flow rates. Isobaric chamber PXs have been assumed due to their better performance [42]. Pressure exchangers from Energy Recovery Inc. (ERI) have been considered. The efficiency $\eta_{PX}$ of the PX (provided by the manufacturer) is defined as depicted in Equation (7):

(7) $$\eta_{PX}=\frac{\sum^{\;}{\left(p\cdot q\right)}_{OUT}}{\sum^{\;}{\left(p\cdot q\right)}_{IN}}=\frac{\left(q_{V,sw}+L\right)\cdot \left(p_{B}-HPDP\right)+\left(q_{V,B}-L\right)\cdot \left(p_{sw}-LPDP\right)}{q_{V,B}\cdot p_{B}+q_{V,sw}\cdot p_{sw}}$$

where $q_{V}$ (m^3^/h) is the flow rate, p (bar) is the pressure, L (m^3^/h) is the lubrication flow (considered null in this work), HPDP (bar) is the high-pressure differential pressure (defined by the manufacturer as the difference of pressure between the brine flow inlet and the seawater flow outlet), LPDP (bar) is the low-pressure differential pressure (the pressure difference between the seawater inlet and the brine flow outlet). In addition, the subscripts *sw* and *B* mean seawater and brine, respectively. An efficiency of 97% has been assumed for all the pressure exchangers. In addition, an HPDP of 0.7 bar and an LPDP of 0.6 bar has been assumed.

#### 2.2.5. Specific Energy Consumption

The specific energy consumption (kWh/m^3^) of the desalination process is determined with Equation (8):

(8) $$\mathrm{SEC}=\frac{\sum^{\;}P_{W,p}-P_{W,T}}{q_{V,perm}}$$

where $P_{W,p}$ (kW) is the power consumption of the pumps, $P_{W,T}$ (kW) is the power production of a Pelton turbine (only in configuration C), and $q_{V,perm}$ (m^3^/h) is the volumetric flow rate of the permeate.

Note that in configuration D, the total SEC is the sum of the SWRO and BWRO SECs weighted by the correspondent flow rates of the permeate:

(9) $$\mathrm{SEC}=\frac{{\mathrm{SEC}}_{RO}\cdot q_{V,p,RO}+{\mathrm{SEC}}_{BW}\cdot q_{V,p,BW}}{q_{V,perm,tot}}$$

The power consumption for each pump i has been calculated with Equation (10):

(10) $$P_{W,p,i}=\frac{q_{V,i}\cdot \Delta p_{i}}{\eta_{s,i}\cdot \eta_{m,i}}$$

where $q_{V,i}$ (m^3^/s), $\Delta p_{i}$ (bar), $\eta_{s,i}$ (-), and $\eta_{m,i}$ (-) are the volumetric flow rates, the pressure gain, the isentropic efficiency, and the mechanical efficiency of the pump i, respectively.

The power production of a Pelton turbine $P_{W,T}$ has been approximated as:

(11) $$P_{W,T}=q_{V}\cdot \Delta p\cdot \eta_{T}$$

where $\eta_{T}$ is the total efficiency of the turbine.

## 3. Results and Discussion

### 3.1. Configurations of the Patent (A, B and C)

**Figure 7:**
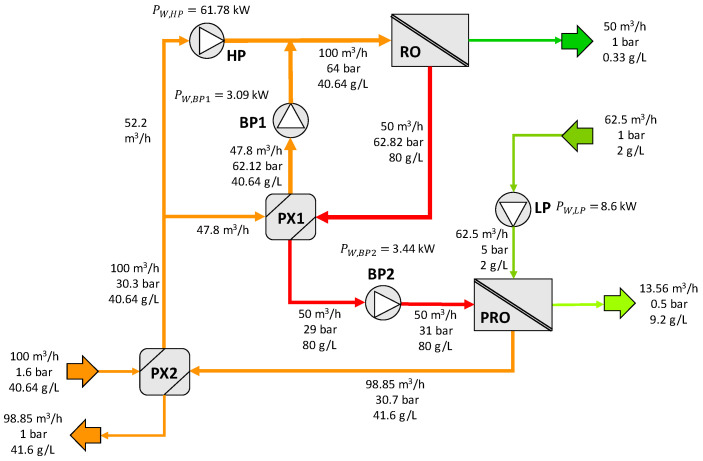
Results for configuration A. HP =high-pressure pump, LP = low-pressure pump, BP = booster pump, PX = pressure exchanger.

Figure 7 shows the results for the first SWRO-PRO configuration proposed in the patent (configuration A). All the streams have been defined by their volumetric flow rate, pressure, and salt concentration. The solving of the pressure exchangers has been conducted assuming a volumetric flow rate of 100 m^3^/h of feed seawater in PX2 and 50 m^3^/h of rejected brine in PX1 (the same as the patent). Taking that into account, the amount of water flow rate transferred in the PRO has been adjusted to match the flow rate needed in PX2, resulting in small differences from the values reported in the patent. This on-design method permits the determination of the amount of membrane required to obtain these conditions. The pumping power consumption has been determined for all the pumps using the mass balance equations together with Equations (7)–(11). The highest power consumption is related to the high-pressure pump (HP) of the SWRO process, which reached a value of 61.78 kW.

**Figure 8:**
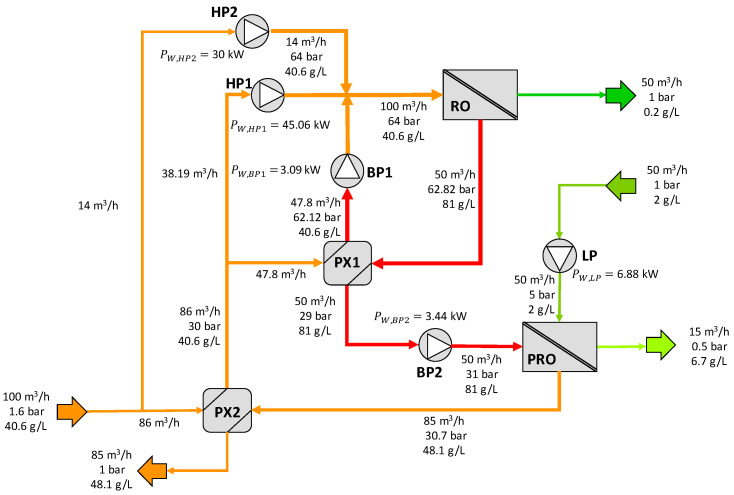
Results for configuration B. HP = high-pressure pump, LP = low-pressure pump, BP = booster ump, PX = pressure exchanger.

The results related to configuration B are depicted in Figure 8. Note that in this layout, there are two high-pressure pumps in the RO, with a power consumption of 45 kW and 30 kW, for HP1 and HP2 pumps, respectively. The increase in the HC mass flow rate in the PRO is 70%.

**Figure 9:**
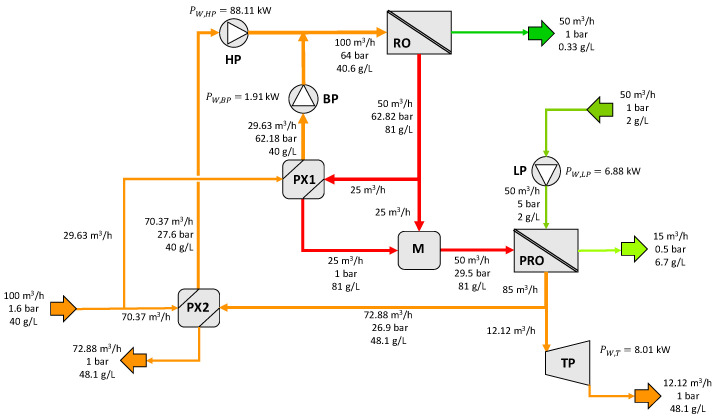
Results for configuration C. HP =high-pressure pump, LP = low-pressure pump, BP = booster pump, PX = pressure exchanger, M = mixer, PT = Pelton turbine.

Figure 9 shows the results for configuration C of the patent where the second booster pump BP2 has been replaced by a mixer and part of the diluted PRO draw solution is sent to a Pelton turbine for power generation.

**Figure 10:**
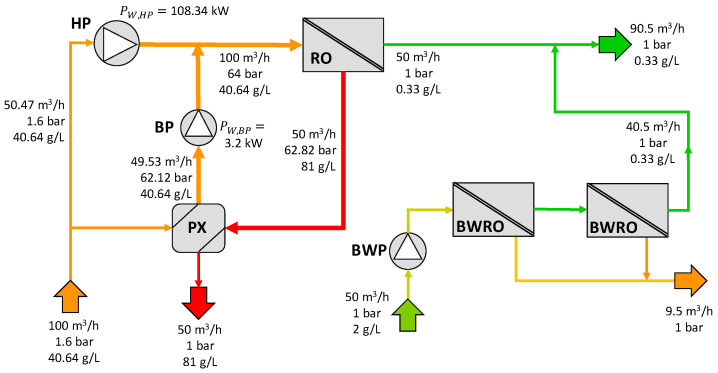
Results for configuration D.

Finally, the determination of the pumping power consumption and permeate volumetric flow in configuration D is presented in Figure 10.

### 3.2. Performance Comparison

A resume of the results obtained for the three configurations presented in the patent (A, B and C) and the base case introduced for the comparison (configuration D) is presented in Table 7.

As it can be seen in Table 7, among the configurations presented in the patent, configuration A results have the lowest SEC (1.54 kWh/m^3^), followed by configurations B and C. In addition, this layout does not have a hydraulic turbine associated, like in the third scheme, which reduces the capital and maintenance costs of the system.

Considering the treatment of industrial wastewater in a low-salinity water source, the actual regulations forbid the reuse of this kind of wastewater. In this case, configuration A is more energy efficient. Configuration A uses this water stream to pressurize the feed seawater through a pressure exchanger after the seawater has been pretreated, thus resulting in an energy consumption decrease. Nevertheless, this kind of energy recovery produces a certain amount of mixing between the streams. To solve the problem of complying with the regulations, it is recommended that a second pressure exchanger (PX2) with a turbocharger will be replaced, which does not include any mixing between the solutions. However, it would also slightly increase the total SEC of the desalination process due to its lower efficiency (~90%) compared to the PX (~95–97%). The best solution, in this case, would be the second configuration of the patent, and replacing the second PX with a turbocharger.

If the low salinity water source is treated with urban wastewater, the actual regulations on non-industrial wastewater reclamation would require two membrane passes of NF, RO, PRO, or FO. This requisite is fulfilled by the two passes BWRO. In this case, the best solution with a lower specific energy consumption would be the conventional SWRO process with the two-pass BWRO. The integration of both systems achieves a specific energy consumption of 1.46 kWh/m^3^. In addition, the scheme out of the patents which would provide the lowest specific energy consumption (1.54 kWh/m^3^) is the first one (A), followed by the second (B) and third (C) configuration (1.75 and 1.78 kWh/m^3^, respectively), with slightly higher values. Compared with the conventional SWRO process (assuming an SEC of 3 kWh/m^3^), a 50% reduction of the SEC is achieved.

The key concept in energy efficiency seems to be the prior compression of the full feed flow of the SWRO desalination process—first configuration (A). This layout exhibits an important advantage regarding its implementation since PX2, BP2, and the PRO subsystem can be placed at the seawater intake infrastructure, thus making it easier for implementation as existing plant retrofitting. Note that the rest of the configuration corresponds to a conventional SWRO plant. On the contrary, layouts B and C are difficult to be adopted by existing plants.

Configuration A achieves an SEC value lower than those of configurations B and C since it avoids the pumping of more seawater feed flow. Indeed, in configuration A, the PRO system replaces the conventional pumping of 100 m^3^/h up to 30.0 bar, in configuration B 86 m^3^/h up to 30 bar, and only 70.26 m^3^/h up to 27.6 bar in configuration C. In the latter design layout, the power production, which is attributable to the expansion of 12.12 m^3^/h at the hydraulic turbine, does not compensate for the said effect.

Furthermore, configuration A is more cost-effective than configuration B, which needs two HPs, and configuration C, which includes a hydraulic turbine. Additionally, it is worth noting that the salinity of the brine discharged is quite similar to that of the seawater feed, thus neglecting the associated environmental impact.

## 4. Conclusions

A performance comparison of the three SWRO-PRO schemes proposed by Sarp et al. [37] for energy recovery in the seawater reverse osmosis desalination application was carried out. This paper proposes a methodology of comparison in which not only the SWRO desalination system is considered but also the treatment of the low-concentration flow of the PRO system.

The proposed hybrid schemes of SWRO-PRO allow for energy consumption reduction in the overall desalination process by recovering part of the energy associated with the salinity gradient between the RO brine and wastewater/brackish water. All three are treated the same volumetric flow rate of seawater (40.6 g/L) and low salinity water (2 g/L). To perform a fair comparison between the arrangements, another scheme using the conventional SWRO and BWRO processes was included in the analysis. As a result, the main design recommendations can be summarized as follows:

The reuse of industrial wastewater is, in general forbidden; therefore, in this case, the use of a BWRO system would be discarded due to the risk of mixing. Configuration A could be used with industrial wastewater if the PX2 were changed by a turbocharger, hence avoiding any mixing in the streams. This configuration is the most energy-efficient (1.54 kWh/m^3^) among the three schemes presented in the patent.

In addition, if the feed water of the PRO unit is treated wastewater, the best solution is the conventional SWRO process with the conventional two-pass BWRO system (configuration D). When adding this external resource to the standalone SWRO base case, the freshwater production increases up to 90.5 m^3^/h, and the SEC reduces significantly (1.46 kWh/m^3^).

Finally, hybrid SWRO-PRO systems are recommended to reduce the specific energy consumption of seawater desalination if an industrial wastewater source with a low osmotic pressure is available. It is worth noting that 1.5 kWh/m^3^ is achievable if seawater feeds are considered with 40.6 g/L at 28 °C and a 50% recovery rate.

## Figures and Tables

**Table 1:** Summary of the literature review on SWRO-PRO articles.

Reference	SEC Reduction *	SWRO RR	Comment
Altaee et al. [24]	−31% (3.3 vs. 4.8 kWh/m^3^)	50%	The layout proposed relies on feed seawater dilution through the PRO system. SWRO feed 45 g/L. Since no energy recovery device is considered, results concerning energy saving in actual SWRO plants cannot be obtained.
Prante et al. [25]	−40% (1.2 vs. 2 kWh/m^3^)	50%	The layout analyzed consists of using the PRO system as an additional energy source that is placed between two conventional energy recovery devices, with scarce information about the pumps. An SEC of 1 kWh/m^3^ could be reached with high-performing membranes, 50% RR.
Achilli et al. [26]	−49.7% (2.64 vs. 5.25 kWh/m^3^)	30%	Three configurations were assessed: standalone SWRO, SWRO-PX (isobaric pressure exchanger), and SWRO-PRO. The latter includes a valve to expand the brine at the SWRO membrane rack prior to the PRO subsystem. Therefore, it should be less energy efficient than that reported in the previous reference. RR of 20% and 30%; SWRO feed 35–37 g/L.
Kim et al. [27]	−50% (1.1 vs. 2.2 kWh/m^3^)	50%	The energy consumption of a PRO-RO hybrid scheme combining wastewater reuse and seawater desalination is analyzed, where the PRO unit was selected to replace an FO module for feedwater dilution before entering the SWRO membrane elements.
Kurihara et al. [28]	−30%	60%	“Mega-ton Water System”: a large-capacity (10^6^ m^3^/d) SWRO process for seawater desalination and wastewater treatment system (Japan). PRO used as an energy recovery system.
Choi et al. [29]	−20% (2.37 vs. 2.96 kWh/m^3^)	40%	A performance and economic analysis of the SWRO-PRO desalination process is presented, using treated wastewater as an FS. Capacity of 100,000 m^3^/d, and a seawater salinity of 32 g/L. The hybrid scheme was found to be competitive with the standalone SWRO desalination process only for a high price of electricity, low price of PRO membranes, and a high-power density.
Senthil and Senthilmurugan [30]	−49.3% (0.842 vs. 1.66 kWh/m^3^)	23%	The simultaneous production of electricity and water is analyzed. They reported a theoretical assessment and optimization of the performance of six different SWRO-PRO hybrid schemes for energy recovery from the SWRO brine. The lowest SEC with respect to the stand-alone was the SWRO-PRO configuration with direct mixing of the draw solution outlet and the SWRO feedwater, using wastewater as the feed of the PRO unit, an FS inlet concentration of 0.1 g/L and production at 0.054 m^3^/s.
Wan and Chung [31]	−49.8% (1.14 vs. 2.27 kWh/m^3^)	50%	The reduction of energy consumption in the SWRO-PRO desalination process is assessed. Three schemes were evaluated: stand-alone SWRO process, SWRO with two pressure exchangers, and SWRO with two pressure exchangers and PRO, with SECs of 4.13, 2.27, and 1.14 kWh/m^3^, respectively. The configuration studied is similar to that analyzed by Prante et al. [25].
Wan and Chung [32]	−49.7% (0.98 vs. 1.95 kWh/m^3^) −48.7% (1.17 vs. 2.28 kWh/m^3^)	25% 50%	Four SWRO hybrid processes with PRO and FO: open-loop PRO-SWRO, closed-loop PRO-SWRO, SWRO + FO dilution of the brine, and seawater dilution FO + SWRO.
Wang et al. [33]	−42.5% (1.57 vs. 2.73 kWh/m^3^) ^a^ −53.5% (1.27 vs. 2.73 kWh/m^3^) ^b^	50%	The impact of the operating temperature on the SEC of the PRO-SWRO system with two ERDs has been evaluated, being the PRO subsystem placed between the ERDs. Increasing the operating temperature from 25 to 50 °C reduces the SEC by 14.41% (0.6 M NaCl solution as the DS) and 17.93% (1.2 M NaCl solution as the DS).
Bargiacchi et al. [34]	−8% (~1.68 vs. 1.831 kWh/m^3^)	35%	The effect of PRO on the decrease in energy consumption in two different SWRO-PRO configurations is evaluated, using both commercial and experimental hollow fiber membranes and two feed-draw pairs: seawater-brine (35–60 g/L) and brackish-brine (5–60 g/L).
Lee et al. [35]	−25% (4.17 vs. 3.13 kWh/m^3^)		Results of the two-year operation of a 240 m^3^/d SWRO-PRO pilot plant in Korea are presented, within the GMVP project [36], aimed to demonstrate the technical feasibility of the PRO process to extract salinity gradient energy from the brine of a desalination plant.

* SEC reduction in SWRO-PRO system compared to standalone SWRO. ^a^ For a SWRO capacity of 1500 m^3^/d. ^b^ and for a SWRO capacity of 150,000 m^3^/d.

**Table 2:** Chemical composition of the feed seawater at 28 °C, pH = 8.1, and with TDS = 40.6 g/L.

Ions	g/L × 10^3^
Potassium (K^+^)	485.34
Sodium (Na^+^)	12,310.05
Magnesium (Mg^2+^)	1571.05
Calcium (Ca^2+^)	487.36
Carbonate (CO_3_^2−^)	32.24
Bicarbonate (HCO_3_^−^)	160.57
Chloride (Cl^−^)	22,398.76
Fluoride (F^−^)	1.39
Sulphate (SO_4_^2−^)	3157.78
Silica (SiO_2_)	1.61
Boron (B)	5.03

**Table 3:** Chemical composition of the feed seawater at 28 °C, pH = 8.1, TDS = 40.6 g/L.

Concept	Value
Feed Flow to Stage 1, m³/h	100
Feed Pressure, bar	64
Flow Factor	0.85
Total Active Area, m²	3719.7
Recovery rate, %	50
Feed Temperature, °C	28
Feed TDS, mg/L	40,635
Average Pass 1 Flux, L/(m^2^·h)	13.44
Osmotic Pressure:	
Feed, bar	29.08
Concentrate, bar	60.75
Element	SW30XLE-440i
#PV	13
#Ele	7
Number of Elements	91
Conc Flow, m³/h	50
Conc Press, bar	62.8
Perm Flow, m³/h	50
Perm Press, bar	1
Perm TDS, g/L × 10^3^	331

**Table 4:** Comparison between the literature values and calculated values in configuration A. Grey highlight denotes input values of the model.

Stream	Patent			This Work		
	$q_{V}$ (m^3^/h)	p (bar)	S (g/L)	$q_{V}$ (m^3^/h)	p (bar)	S (g/L)
SWRO brine (HC in)	50	31	80	50	31	80
Diluted brine (HC out)	100	30.7	40	103	30.7	40
Feed in (LC in)	62.5	5	2	62.5	5	2
Feed out (LC out)	12.5	0.5	6.7	9.5	0.5	13

**Table 5:** Comparison between the literature values and calculated values in configuration B. Grey highlight denotes input values of the model.

Stream	Patent			This Work		
	$q_{V}$ (m^3^/h)	p (bar)	S (g/L)	$q_{V}$ (m^3^/h)	p (bar)	S (g/L)
SWRO brine (HC in)	50	31	80	50	31	80
Diluted brine (HC out)	85	30.7	47	87	30.7	47
Feed in (LC in)	50	5	2	50	5	2
Feed out (LC out)	15	0.5	6.7	12.9	0.5	7.7

**Table 6:** Comparison between the literature values and calculated values in configuration C. Grey highlight denotes input values of the model.

Stream	Patent			This Work		
	$q_{V}$ (m^3^/h)	p (bar)	S (g/L)	$q_{V}$ (m^3^/h)	p (bar)	S (g/L)
SWRO brine (HC in)	50	29.5	80	50	29.5	80
Diluted brine (HC out)	85	29	47	87	29	47
Feed in (LC in)	50	5	2	50	5	2
Feed out (LC out)	15	0.5	6.7	12.9	0.5	7.7

**Table 7:** Comparison between the results obtained in the configurations A, B, C, and the base case (D).

Concept		Config. A	Config. B	Config. C	Config. D
System		SWRO-PRO	SWRO-PRO	SWRO-PRO	SWRO-BWRO
Feed flow rate (m^3^/h), 40 g/L		100	100	100	100
Permeate (m^3^/h), 0.32 g/L		50	50	50	90.5
Recovery rate (%)		50	50	50	50% SWRO 81% BWRO
PRO FS	Vol. flow (m^3^/h)	62.5	50	50	50
	Salinity (g/L)	2	2	2	2
	Pressure (bar)	1	1	1	1
Pumping power (kW)		76.9	87.48	96.9	111.58
Pelton turbine (kW)		n/a	n/a	−8.01	n/a
BWRO SEC (kWh/m^3^)		n/a	n/a	n/a	0.5
Total SEC (kWh/m^3^)		1.54	1.75	1.78	1.46 *

* The total SEC is determined by weighting each contribution, taking into account the different flow rates of permeate.

## Data Availability

Not applicable.
